# Socio-demographic and lifestyle determinants of dietary patterns in French-speaking Switzerland, 2009–2012

**DOI:** 10.1186/s12889-018-5045-1

**Published:** 2018-01-12

**Authors:** Pedro Marques-Vidal, Gérard Waeber, Peter Vollenweider, Idris Guessous

**Affiliations:** 1 Department of Medicine, Internal Medicine, Lausanne University Hospital (CHUV), Office BH10-642, Rue du Bugnon 46, 1011 Lausanne, Switzerland; 2 Unit of population epidemiology, Division of primary care medicine, Department of community medicine, primary care and emergency medicine, Geneva university hospitals, Geneva, Switzerland; 3 Department of ambulatory care and community medicine, University of Lausanne, Lausanne, Switzerland

**Keywords:** Dietary patterns, Education, Migrants, Obesity, Population-based sample, Switzerland

## Abstract

**Background:**

Food intake is a complex behaviour which can be assessed using dietary patterns. Our aim was to characterize dietary patterns and associated factors in French-speaking Switzerland.

**Methods:**

Cross-sectional study conducted between 2009 and 2012 in the city of Lausanne, Switzerland, including 4372 participants (54% women, 57.3 ± 10.3 years). Food consumption was assessed using a validated food frequency questionnaire. Dietary patterns were assessed by principal components analysis.

**Results:**

Three patterns were identified: “Meat & fries”; “Fruits & Vegetables” and “Fatty & sugary”. The “Meat & fries” pattern showed the strongest correlations with total and animal protein and cholesterol carbohydrates, dietary fibre and calcium. The “Fruits & Vegetables” pattern showed the strongest correlations with dietary fibre, carotene and vitamin D. The “Fatty & sugary” pattern showed the strongest correlations with total energy and saturated fat. On multivariate analysis, male gender, low educational level and sedentary status were positively associated with the “Meat & fries” and the “Fatty & sugary” patterns, and negatively associated with the “Fruits & Vegetables” pattern. Increasing age was inversely associated with the “Meat & fries” pattern; smoking status was inversely associated with the “Fruits & Vegetables” pattern. Being born in Portugal or Spain was positively associated with the “Meat & fries” and the “Fruits & Vegetables” patterns. Increasing body mass index was positively associated with the “Meat & fries” pattern and inversely associated with the “Fatty & sugary” pattern.

**Conclusions:**

Three dietary patterns, one healthy and two unhealthy, were identified in the Swiss population. Several associated modifiable behaviours were identified; the information on socio- demographic determinants allows targeting of the most vulnerable groups in the context of public health interventions.

**Electronic supplementary material:**

The online version of this article (10.1186/s12889-018-5045-1) contains supplementary material, which is available to authorized users.

## Background

Dietary intake is one of the major determinants of health, and it has been repeatedly shown that improving dietary intake leads to an improvement in morbidity and mortality [1]. Dietary intake is a complex behaviour, which cannot be reduced to the consumption of single types of foods or nutrients [2, 3]. Indeed, the variety of foods, nutrients and their interactions considerably complicate the analysis of the associations between individual foods or nutrients and diseases. Hence, multivariate, dimension-reducing approaches such as dietary patterns have been proposed. Dietary patterns could resolve concerns about food and nutrient interactions and provide a more accurate picture of an individual’s dietary behaviour [2, 3]. Dietary patterns have been suggested to be advantageous over individual foods and nutrients regarding the associations between diet and chronic diseases such as diabetes [4]. Furthermore, dietary patterns provide the background to identify specific food combinations that are either protective or deleterious, thus fostering further research regarding individual foods and dietary guidelines [5]. Dietary patterns are also easier to apply in public health policies, as they correspond to “foods that are actually consumed in various characteristic combinations” [6]. Indeed, several individual, lifestyle and socio-demographic factors associated with dietary patterns have been identified: age and education are positively associated with a healthy dietary pattern (mainly characterized by a high intake of fruits, vegetables or fish) [7, 8], while male gender is usually associated with more unhealthy patterns (characterized by high intake of fat, red meat or convenience foods) [7]. Identification of groups with the (un) healthier dietary patterns would allow better public health policies regarding diet [9].

Previous studies conducted in Switzerland [10, 11] assessed differences in single foods or nutrients between socio-demographic and socioeconomic groups. A study in Geneva assessed trends for dietary patterns [12], and it would be of interest if such patterns could be replicated in another Swiss city using the same methodology of data collection. Hence, we aimed to assess dietary patterns and their main determinants in a cross-sectional, population-based sample in Switzerland.

## Methods

### The Cohorte Lausannoise (CoLaus) study.

The CoLaus study is a population-based study assessing the clinical, biological and genetic determinants of cardiovascular disease in the city of Lausanne, Switzerland. Its aims and sampling strategy have been reported previously [13]. The source population was defined as all subjects aged between 35 and 75 years registered in the population register of the city, which also includes information on age and sex. A simple, non-stratified random sample of 19,830 subjects (corresponding to 35% of the source population) was drawn and the selected subjects were invited to participate. The following inclusion criteria were applied: (a) written informed consent; (b) willingness to take part in the examination and to provide blood samples.

The baseline study was conducted between 2003 and 2006 and the first follow-up visit was conducted between April 2009 and September 2012 and included all participants willing to be re-contacted. At follow-up, participants attended a single visit, which included an interview, a dietary assessment, a physical exam, and blood and urine collections in the fasting state. For this study, only data from the follow-up examination was used as dietary intake assessment was first introduced at this time point.

### Socio-demographic and anthropometric data

Age (range: 41–79 years) was categorized into 10-year age groups: 40–49; 50–59; 60–69 and 70–79. Educational level was categorized as low (primary), middle (apprenticeship or secondary school) and high (university). Country of birth was categorized into 6 groups: Switzerland, the four most common countries (providing at least 100 participants) including France, Italy, Portugal and Spain, and other. Analysis according to country of birth was considered as important as a previous study showed considerable differences in dietary intake between these groups [14]. Smoking status was defined as never, former (irrespective of the time since quitting) and current (irrespective of the amount smoked). Body weight and height were measured using standard procedures [13] and body mass index (BMI) was defined as weight (kg)/height(m)^2^. Overweight was defined as 25 ≤ BMI < 30 kg/m^2^ and obesity as BMI ≥ 30 kg/m^2^.

### Physical activity assessment

Physical activity was assessed by a questionnaire [15] validated in the population of Geneva. This self-reported questionnaire assesses the type and duration of 70 kinds of (non)professional activities and sports during the previous week. Sedentary status was defined as spending more than 90% of the daily energy in activities below moderate- and high-intensity (defined as requiring at least 4 times the basal metabolic rate, BMR) [16, 17]. BMR multiples are close to Metabolic Equivalent of Task (MET) multiples, although MET multiples do not take into account participant sex, age or height.

### Dietary assessment

Dietary intake was assessed using a self-administered, semi-quantitative food frequency questionnaire (FFQ) which also includes portion size [18]. This FFQ was validated in the Geneva population [18, 19]. Briefly, this FFQ assesses the dietary intake of the previous 4 weeks and consists of 97 different food items accounting for more than 90% of the intake of calories, proteins, fat, carbohydrates, alcohol, cholesterol, vitamin D and retinol, and 85% of fibre, carotene and iron. To our knowledge, there is no FFQ (validated or not) assessing dietary intake for the whole year in Switzerland. Hence, this FFQ provides the best dietary assessment currently available for French speaking Switzerland. For each item, consumption frequencies ranging from “less than once during the last 4 weeks” to “2 or more times per day” were provided, and the participants also indicated the average serving size (smaller, equal or bigger) compared to a reference size. Each participant brought along her/his filled-in FFQ, which was checked for completion by trained interviewers on the day of the visit. Dietary patterns were assessed using consumption frequencies, defined as “never these last 4 weeks” = 0; “once/month” = 1/28; “2–3/month” = 2.5/28; “1–2/week” = 1.5/7; “3–4 times/week” = 3.5/7; “once/day” = 1 and “2+/day” = 2.5. The 97 items were then grouped into 40 food and nutrient groups, including vitamin and food supplements (Additional file 1: Table S1). Conversion into nutrients was performed based on the French CIQUAL food composition table [20] taking into account portion size. The use of a French food composition table was motivated by the fact that no adequate Swiss food composition table existed when the FFQ was constructed and validated. Reference portions were defined by the use of common measures such as “one slice” (of bread); “one yogurt cup” “(for peas or berries)”; “one tablespoon”; “one serving” (for tomatoes or bananas) or “one glass” (of water or of wine, as size depends on the type of beverage). The reference portion was defined as the median of portion size distribution in the validation paper, and the “smaller” and “bigger” portions were defined as the first and the third quartiles of the distribution [21]. Total energy intake (TEI) was computed and alcohol consumption was included in this calculation.

Participants were considered to be on a diet if they responded positively to the question “are you currently on a diet”, irrespective of the type of diet considered (for slimming, diabetes, high cholesterol, other).

### Exclusion criteria

Participants were excluded if they presented at least one of the following characteristics: 1) No FFQ completed; 2) Less than 30 items consumed according to the completed FFQ; 3) No data for smoking or education.

### Statistical analysis

Statistical analyses were performed using Stata version 14.2 for windows (Stata Corp, College Station, Texas, USA). Descriptive results were expressed as number of participants (percentage) or as average ± standard deviation. Bivariate analyses were performed using chi-square for categorical variables and Student’s t-test or analysis of variance for continuous variables.

Dietary patterns were assessed by principal components analysis (PCA) with varimax rotation as done in previous studies [7, 22–24]. The Kaiser-Meyer-Olkin (KMO) and the Bartlett test for sphericity were applied to assess the appropriateness of applying PCA to the dataset. The Bartlett test compares the correlation matrix between the different items to be included in the PCA to the identity matrix. A non-significant Bartlett test indicates that the variables are highly correlated and that information compression using PCA is not useful. The KMO was 0.755, which was above the suggested minimum of 0.5 [25] and comparable to values reported in the literature [7, 24]. The Bartlett test for sphericity yielded a *p*-value of < 0.0001. Hence, both KMO and the Bartlett tests indicated that the data were suitable for PCA.

The number of dietary patterns to be retained was determined based on the same criteria as described by others [8, 22], namely 1) analysis of the scree plot; 2) an eigenvalue higher than one and 3) the interpretability of the dietary pattern. For interpretation purposes, varimax rotation was performed. Items with absolute factor loading > 0.30 were considered to characterize the dietary patterns [1], although all items were used to calculate dietary pattern scores. As suggested previously [24], the associations between dietary patterns and nutrients were assessed using Pearson correlations and corresponding 95% confidence intervals, applying Fisher’s z transformation and the **corrci** command of Stata. Bivariate comparison of correlation coefficients was performed using Steiger’s method and using the **corcor** command of Stata.

Dietary patterns were categorized into quintiles and the distributions of individual and behaviour factors and dietary patterns were compared between the highest quintile and the other four, a method also used elsewhere [7, 23, 24]. Multivariable analysis was performed using Poisson regression for highest quintile vs. the others, as previously reported [7]. Poisson regression was preferred to logistic regression because the outcome of interest was not a rare event (20%), and using logistic regression would overestimate the associations [26]. All variables associated with at least one dietary pattern in the bivariate analysis were included in the multivariate model. Results were expressed as prevalence rate ratio (PRR) and 95% confidence interval. Tests for trends were assessed using the **contrast q.** command of Stata.

As complete physical activity data was only available for a limited number of participants, the initial separate analyses were performed, including or not the sedentary status in the multivariable model. Other sensitivity analyses were performed: 1) excluding participants with a total energy intake < 850 or > 4500 kcal/day [27]; 2) using food pattern scores as continuous variables. For the latter case, analyses were performed using analysis of variance. Tests for trends were assessed using the **contrast q.** function of Stata. Statistical significance was considered for a two-sided test *p* < 0.05.

## Result

### Selection procedure and characteristics of participants

**Fig. 1:**
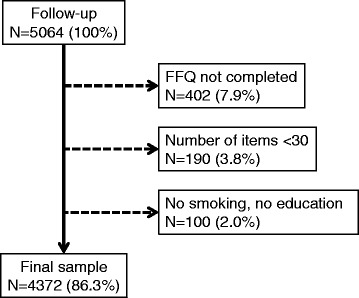
Selection procedure of the participants of the CoLaus study, 2009–2012, Lausanne, Switzerland

Of the 5064 participants available at follow-up, 692 (13.7%) were excluded. The reasons for the exclusion are summarized in Fig. 1 and the main characteristics of participants included and excluded are summarized in Additional file 1: Table S2. Excluded participants were older, with a higher BMI, and were more frequently born outside Switzerland, with lower education, current smokers, sedentary and obese. Thus, the analysis included 4372 participants, 3936 (90%) of whom had data for physical activity.

### Dietary patterns

The results of the principal components analysis are summarized in Table 1. Three dietary patterns were identified, explaining 20.9% of the overall variance. The first dietary pattern was named “Meat & fries” (unhealthy) and had high loadings for all kinds of meat and French fries. The second dietary pattern was named “Fruits & Vegetables” (healthy) and had high loadings for fruits and vegetables. The third dietary pattern was named “Fatty & sugary” (unhealthy) and had high loadings for hard fats (i.e. butter, margarine), pastries and sugar-rich foods (Table 1).

**Table 1:** Factor loadings derived from principal component analysis and percentage of total variance explained for the three dietary patterns identified, 4372 participants of the CoLaus study, 2009–2012, Lausanne, Switzerland

Food group	Item	Meat & fries	Fruits & vegetables	Fatty & sugary
Dairy	Full fat or semi-skimmed dairy products	−0.076	0.074	0.207
	Low fat dairy products	0.007	0.166	−0.124
	Whole milk cheese	−0.035	0.091	0.259
Bread and cereals	White bread	0.091	−0.137	0.297
	Wholemeal bread	−0.092	0.258	−0.008
	Breakfast cereals	−0.091	0.195	−0.030
	Toasts, crackers	−0.024	0.125	−0.024
Meat	Red meat	**0.421**	0.008	0.015
	Poultry	**0.389**	0.104	−0.098
	Processed meat	**0.423**	−0.039	0.047
	Liver	**0.419**	0.054	−0.099
Fish	Oily fish	0.118	0.240	−0.148
	Canned or fried fish	0.151	0.106	0.017
	Lean fish & seafood	0.069	0.287	−0.116
Vegetables	Vegetables	0.154	**0.366**	0.004
	Boiled potatoes	0.091	0.179	0.120
	French fries	**0.344**	−0.135	0.063
Pasta, other	Sauces (any)	0.041	0.242	0.116
	Cafeteria foods	0.138	−0.032	0.223
	Starchy foods	0.108	0.142	0.159
	Eggs	0.045	0.160	0.074
	Tofu	−0.049	0.147	−0.030
Fruit	Fresh fruit or juice	−0.085	**0.358**	0.044
	Canned fruit	0.031	0.044	0.126
Fats	Low-cal fat products	−0.013	0.096	0.010
	Hard fats	−0.004	0.026	**0.374**
	Olive oil	−0.043	0.269	0.093
	Other vegetable oils	0.037	0.090	0.201
Pastries and sweets	Bakery	−0.010	−0.029	**0.345**
	Chocolate	−0.086	0.022	**0.447**
	Sugar substitutes	−0.005	0.078	−0.083
Vitamins, supplements	Vitamin supplements	−0.047	0.116	−0.034
	Other supplements	−0.040	0.118	−0.106
Drinks	Sodas	0.101	−0.061	0.206
	Tea & coffee	−0.106	0.170	0.141
	Water	−0.040	0.176	0.007
	Alcoholic drinks	0.100	−0.154	0.081
	% variance explained	8.1	7.2	5.6

Factor loadings with absolute values > 0.300 were used to characterize the dietary pattern and are indicated in bold

The Pearson correlations, with 95% confidence intervals, between the three dietary pattern scores and selected macro- and micronutrients are provided in Additional file 1: Table S3. Almost all correlations were statistically significant. The “Meat & fries” pattern showed the strongest positive correlations with total and animal protein, cholesterol and iron, and inverse correlations with total carbohydrates, dietary fibre and calcium. The “Fruits & Vegetables” pattern showed the strongest positive correlations with vegetable protein, dietary fibre, carotene and vitamin D, the weakest positive correlations with cholesterol, and a negative correlation with alcohol. The “Fatty & sugary” pattern showed the strongest positive correlations with total energy intake and saturated fat, and the weakest positive correlation with vitamin D (Additional file 1: Table S3).

### Factors associated with dietary patterns

Bivariate and multivariate associations between participants’ characteristics and the three dietary patterns identified are summarized in Tables 2 and 3, respectively. In multivariate analysis, male gender was positively associated with the “Meat & fries” and the “Fatty & sugary” patterns and inversely associated with the “Fruits & Vegetables” pattern. Increasing age was inversely associated with the “Meat & fries” pattern. Being born in Portugal or Spain was positively associated with the “Meat & fries” and the “Fruits & Vegetables” patterns. Lower educational level was positively associated with the “Meat & fries” pattern and inversely associated with the “Fruits & Vegetables” pattern. Smoking status was inversely associated with the “Fruits & Vegetables” pattern; being on a diet was positively associated with the “Fruits & Vegetables” pattern and inversely associated with the “Meat & fries” and the “Fatty & sugary” patterns. Increased BMI was positively associated with the “Meat & fries” pattern and inversely associated with the “Fatty & sugary” pattern.

**Table 2:** Distribution of sociodemographic and lifestyle characteristics across highest and lowest quintiles of dietary patterns scores identified among 4372 participants the CoLaus study, 2009–2012, Lausanne, Switzerland

	Meat & fries		Fruits & vegetables		Fatty & sugary	
	Q_1–4_	Q_5_	Q_1–4_	Q_5_	Q_1–4_	Q_5_
Gender						
Woman	2034 (86.2)	325 (13.8)	1742 (73.8)	617 (26.2)	1952 (82.8)	407 (17.3)
Man	1465 (72.7)	549 (27.3)	1757 (87.2)	257 (12.8)	1547 (76.8)	467 (23.2)
*p*-value	< 0.001		< 0.001		< 0.001	
Age group (years)						
40–49	961 (75.1)	318 (24.9)	1057 (82.6)	222 (17.4)	1018 (79.6)	261 (20.4)
50–59	1050 (78.0)	297 (22.0)	1082 (80.3)	265 (19.7)	1072 (79.6)	275 (20.4)
60–69	991 (85.1)	173 (14.9)	888 (76.3)	276 (23.7)	964 (82.8)	200 (17.2)
70–79	497 (85.3)	86 (14.7)	472 (81.0)	111 (19.0)	445 (76.3)	138 (23.7)
*p*-value	< 0.001		0.001		0.012	
Country of birth						
Switzerland	2387 (83.9)	458 (16.1)	2329 (81.9)	516 (18.1)	2286 (80.3)	559 (19.7)
France	222 (76.6)	68 (23.5)	225 (77.6)	65 (22.4)	215 (74.1)	75 (25.9)
Italy	173 (81.6)	39 (18.4)	173 (81.6)	39 (18.4)	167 (78.8)	45 (21.2)
Portugal	108 (54.0)	92 (46.0)	145 (72.5)	55 (27.5)	163 (81.5)	37 (18.5)
Spain	85 (63.4)	49 (36.6)	101 (75.4)	33 (24.6)	110 (82.1)	24 (17.9)
Other	524 (75.7)	168 (24.3)	526 (76.0)	166 (24.0)	558 (80.6)	134 (19.4)
*p*-value	< 0.001		< 0.001		0.188	
Education						
University	787 (80.1)	195 (19.9)	745 (75.9)	237 (24.1)	789 (80.4)	193 (19.7)
High school	952 (81.7)	214 (18.3)	914 (78.4)	252 (21.6)	940 (80.6)	226 (19.4)
Apprenticeship	1279 (81.9)	282 (18.1)	1311 (84.0)	250 (16.0)	1241 (79.5)	320 (20.5)
Primary	481 (72.4)	183 (27.6)	529 (79.7)	135 (20.3)	529 (79.7)	135 (20.3)
*p*-value	< 0.001		0.001		0.887	
Smoking status						
Never	1457 (80.6)	350 (19.4)	1410 (78.0)	397 (22.0)	1480 (81.9)	327 (18.1)
Former	1354 (81.4)	309 (18.6)	1299 (78.1)	364 (21.9)	1321 (79.4)	342 (20.6)
Current	688 (76.2)	215 (23.8)	790 (87.5)	113 (12.5)	698 (77.3)	205 (22.7)
*p*-value	0.005		< 0.001		0.014	
On a diet						
No	2361 (78.9)	632 (21.1)	2478 (82.8)	515 (17.2)	2319 (77.5)	674 (22.5)
Yes	1138 (82.5)	242 (17.5)	1021 (74)	359 (26)	1180 (85.5)	200 (14.5)
*p*-value	0.006		< 0.001		< 0.001	
BMI categories						
Normal	1657 (83.7)	322 (16.3)	1549 (78.3)	430 (21.7)	1555 (78.6)	424 (21.4)
Overweight	1305 (77.5)	378 (22.5)	1368 (81.3)	315 (18.7)	1345 (79.9)	338 (20.1)
Obese	537 (75.5)	174 (24.5)	582 (81.9)	129 (18.1)	599 (84.3)	112 (15.8)
*p*-value	< 0.001		0.031		0.005	
Sedentary						
No	1379 (81.2)	319 (18.8)	1317 (77.6)	381 (22.4)	1362 (80.2)	336 (19.8)
Yes	1778 (79.4)	461 (20.6)	1814 (81.0)	425 (19.0)	1772 (79.1)	467 (20.9)
*p*-value	0.160		0.008		0.409	

*BMI* body mass index, *Q*_*1–4*_ first to fourth quintiles, *Q*_*5*_ fifth quintile. Analysis performed on 4372 participants, except for sedentary status (*N* = 3936). Results are expressed as number of participants and (row percentage). Statistical analysis performed using chi-square

**Table 3:** Multivariable analysis of the associations between personal and behavioural factors and being in the highest quintile of the three dietary patterns identified, 4372 participants of the CoLaus study, 2009–2012, Lausanne, Switzerland

	Meat & fries	Fruits & vegetables	Fatty & sugary
Gender			
Woman	1 (ref.)	1 (ref.)	1 (ref.)
Man	1.89 (1.64–2.19)	0.48 (0.42–0.56)	1.37 (1.19–1.57)
*p*-value	< 0.001	< 0.001	< 0.001
Age group			
40–49	1 (ref.)	1 (ref.)	1 (ref.)
50–59	0.92 (0.78–1.08)	1.15 (0.96–1.38)	1.03 (0.87–1.22)
60–69	0.71 (0.59–0.87)	1.36 (1.13–1.64)	0.93 (0.77–1.13)
70–79	0.71 (0.55–0.91)	1.15 (0.91–1.47)	1.28 (1.03–1.58)
*p-value for trend*	0.001	0.116	0.072
Country of birth			
Switzerland	1 (ref.)	1 (ref.)	1 (ref.)
France	1.51 (1.16–1.95) **	1.07 (0.83–1.40)	1.39 (1.09–1.77) **
Italy	0.98 (0.70–1.36)	1.19 (0.85–1.65)	1.04 (0.76–1.42)
Portugal	2.04 (1.56–2.67) ***	2.05 (1.49–2.80) ***	0.90 (0.62–1.30)
Spain	1.93 (1.42–2.63) ***	1.50 (1.05–2.16) *	0.92 (0.60–1.39)
Other	1.52 (1.26–1.83) ***	1.13 (0.94–1.35)	1.09 (0.90–1.33)
Education			
University	1 (ref.)	1 (ref.)	1 (ref.)
High school	1.00 (0.82–1.22)	0.81 (0.68–0.97)	1.06 (0.87–1.29)
Apprenticeship	1.07 (0.88–1.30)	0.61 (0.50–0.74)	1.15 (0.96–1.39)
Primary	1.26 (0.99–1.60)	0.61 (0.48–0.78)	1.22 (0.96–1.57)
*p-value for trend*	0.045	< 0.001	0.077
Smoking status			
Never	1 (ref.)	1 (ref.)	1 (ref.)
Former	0.92 (0.79–1.07)	1.08 (0.93–1.25)	1.13 (0.97–1.31)
Current	1.13 (0.95–1.34)	0.64 (0.52–0.79)	1.19 (0.99–1.41)
*p-value for trend*	0.162	< 0.001	0.059
On a diet			
No	1 (ref.)	1 (ref.)	1 (ref.)
Yes	0.85 (0.73–0.99)	1.44 (1.25–1.65)	0.66 (0.56–0.78)
p-value	0.032	< 0.001	< 0.001
BMI categories			
Normal	1 (ref.)	1 (ref.)	1 (ref.)
Overweight	1.21 (1.03–1.41)	0.94 (0.81–1.10)	0.89 (0.77–1.03)
Obese	1.43 (1.18–1.74)	0.85 (0.69–1.04)	0.74 (0.60–0.92)
*p-value for trend*	< 0.001	0.120	0.006

Analysis performed on 4372 participants. Results are expressed as prevalence rate ratios and (95% confidence interval) of being in the last quintile relative to the other four. Statistical analysis performed using Poisson regression adjusting for the variables listed in the tables. All variables were simultaneously included in the model. For country of birth, significant associations are indicated as follows: *, *p* < 0.05; **, *p* < 0.01; ***, *p* < 0.001

### Sensitivity analyses

The results after excluding participants without data on sedentary status are summarized in Additional file 1: Tables S5 (for highest quintile vs. others) and Additional file 1: Tables S6 (for dietary pattern scores as continuous variables). The results after excluding participants with extreme reported energy intakes are summarized in Additional file 1: Tables S7 (for highest quintile vs. others) and Additional file 1: Tables S8 (for dietary pattern scores as continuous variables). The results after excluding participants with extreme reported energy intakes or without data for sedentary status are summarized in Additional file 1: Tables S9 (for highest quintile vs. others) and Additional file 1: Tables S10 (for dietary pattern scores as continuous variables). Overall, the findings were similar to those reported for the whole sample, with sedentary status being positively associated with the “Meat & fries” pattern (and to a lesser degree to the “Fatty & sugary” pattern) and inversely associated with the “Fruits and Vegetables” pattern.

## Discussion

This is one of the few studies to characterize empirically-derived dietary patterns in the French-speaking Swiss population. Three patterns were identified, and several socio-demographic and lifestyle factors were found to be associated with them.

### Dietary patterns

Three patterns were identified; based on the dietary guidelines of the International Agency for Research on Cancer [28], one was termed as healthy (“Fruits & Vegetables”) and two as unhealthy (“Meat & fries” and “Fatty & sugary”). The three patterns explained 20.9% of the overall variance in food consumption; this relatively low percentage of explained variance is likely due to the large number of food groups included in the PCA [3] but is similar to other studies [7, 24], including one conducted in Geneva using the same FFQ [12]. Also, the patterns identified in this study were almost identical to those reported in Geneva [12] and comparable to dietary patterns reported in other countries. For instance, the “Meat & fries” pattern was identical to one described in Puerto Rico [29] and involved several components shared with the “Western” pattern identified in Sweden [24]. The “Fruits & Vegetables” pattern shared the same components as the “Healthy” pattern described in a Swedish study [30] and the “Olive oil and vegetables” identified in Italy [31]. Finally, the “Fatty & sugary” pattern involved most components of the “convenience foods” identified in France [8], the “Eggs and sweets” identified in Italy [31] some components of the “Western” pattern identified in Brazil [32] and the “Continental” pattern identified in Sweden [24]. Overall, our results suggest that, notwithstanding different dietary assessment methods, the dietary patterns identified in this study share several characteristics with other patterns identified in other settings.

### Factors associated with dietary patterns

Women had higher PRRs and scores for the “Fruits & Vegetables” pattern and lower PRRs and scores for the “Meat & fries” and the “Fatty & sugary” patterns, a finding in agreement with the literature [7, 33]. These findings confirm the higher importance of diet for women compared to men, a finding also reported when assessing compliance to dietary recommendations [11].

Elderly subjects had higher PRRs and scores for the “Fruits & Vegetables” pattern and lower PRRs and scores for the “Meat & fries” pattern, a finding in agreement with the literature [33]. The “Meat & fries” dietary pattern was associated with an increasing allostatic load [29] risk for diabetes [34] and acute myocardial infarction [35], while patterns such as the “Fruits & Vegetables” pattern have been shown to be protective [35]. Thus, the unhealthy dietary patterns in younger participants might favour the increase in the prevalence of obesity and cardiovascular risk factors (namely diabetes) in this group [36]. Conversely, the higher PRR and scores for the “Fatty & sugary” pattern among the eldest group could be due to several factors including a decreased sense of taste [37] or a decreased financial capacity forcing older people to buy less expensive, more sugar and fat rich foods [38].

Being born in Portugal or Spain was positively associated with both the “Meat & fries” and the “Fruits & Vegetables” patterns. A possible explanation is that migrants from these countries improve their wealth when working in Switzerland, making them buy more meat (a marker of wealth) while maintaining some of their traditional dietary patterns (i.e. Fruits & Vegetables). Indeed, a previous study conducted in Portugal showed that the improvement in overall wealth after joining the EU in the nineties led to a considerable change in diet, shifting from a south European to a more Westernized, protein-rich diet [39]. Conversely, as the “Meat & fries” pattern includes all types of meat, it was not possible to assess if the increase in meat was related to the most expensive parts of meat (beef) or to the cheaper ones such as processed meat.

Highly educated participants had higher PRRs and scores for the “Fruits & Vegetables” pattern and lower PRRs and scores for the “Meat & fries” and to a lesser degree for the “Fatty & sugary” patterns, a finding repeatedly reported in the literature, [7, 30, 33]. A likely explanation is that highly educated people are more compliant with dietary recommendations [11], and tend to have a higher income enabling them to buy more fruits and vegetables than less educated people [38].

Current smokers had lower PRRs and scores for the “Fruits & Vegetables” pattern, a finding also reported previously [30, 40]. Conversely, no significant differences were found for the “Meat & fries” and the “Fatty & sugary” patterns, suggesting that current smoking selectively impairs the consumption of specific foods. Possible explanations include a lower compliance to dietary recommendations [11], tobacco-induced changes in sensory system, gustatory impairment as a consequence of heavy smoking [41] and decreased olfactory capacity [42], making smokers select foods with stronger flavours (i.e. more salty).

Participants reporting being on a diet had higher PRRs and scores for the “Fruits & Vegetables” pattern and lower PRRs and scores for the “Meat & fries” and the “Fatty & sugary” patterns, a finding also reported elsewhere [30] and suggestive for the increased awareness on the importance of dietary intake. Due to the large variation in the type of diets, it was not possible to precisely assess associations between each type of diet and the different dietary patterns.

Sedentary participants had lower PRR for the “Fruits & Vegetables” pattern and tended to present higher PRRs for the unhealthy ones; when the analysis was based on dietary pattern scores, clear differences were found; sedentary participants scoring had higher scores in the “Meat & fries” and the “Fatty & sugary” patterns and lower ones in the “Fruits & Vegetables” pattern. Such findings have been repeatedly reported in the literature [30, 40]. Overall, our results reinforce the fact that dietary patterns are closely related to several lifestyle characteristics.

Obese participants had lower PRRs for the “Fatty & sugary” pattern and higher PRRs for the “Meat & fries” pattern, and these associations persisted after excluding participants reporting to be on a diet. The most likely explanation is a reporting bias; obese participants may underreport the intake of foods which they consider as obesogenic. Interestingly, a negative association between BMI and the “Fatty & sugary” pattern was observed, but a positive association with the “Meat & fries” was found. This former association might be due to the fact that most people do not consider meat as obesogenic, although the increased consumption of meat has been shown to be associated with obesity [43]. Finally, a significant negative association between BMI categories and the “Fruits & Vegetables” pattern was found after excluding participants with extreme energy intakes, a finding also reported previously [23]. Overall, our results indicate that increased BMI is associated with unhealthy dietary patterns, and this association might be partly blurred by reporting bias.

### Impact for dietary policies

Several modifiable behaviours were associated with dietary patterns, allowing for a better targeting of the most vulnerable groups in the context of public health interventions, although such modifications have been questioned [44]. For instance, smokers should be urged to increase vegetable consumption, while the promotion of physical activity would allow tackling both sedentary status and the associated dietary patterns.

### Strengths and limitations

This study has several limitations. First, the cross-sectional setting of the study only allows establishing associations, and no causal inferences can be drawn. Second, excluded participants differed significantly from those whom the dietary patterns were computed accordingly; hence, dietary patterns were derived from a healthier sample and might not fully represent the true dietary patterns in the general population. Still, the patterns identified were similar to those reported in other studies, and could serve as a foundation for future studies on dietary behaviours in French-speaking Switzerland. Third, only urban citizens were queried, and we have no information regarding dietary patterns of rural inhabitants. Still, according to the Swiss federal office of statistics, in 2014, 84% of the Swiss population lived in an urban setting [45], so our results apply to the majority of the French-speaking Swiss population. Fourth, portion size was self-reported and might have been misevaluated by the participants; still, this is a common issue among self-reported dietary intake and it has been shown that dietary patterns do not change significantly when input variable quantification changes [46]. Finally, the study was conducted in a French-speaking canton; as Switzerland is a multilingual country, it is possible that dietary behaviours in German or Italian -speaking regions may be different, but no data is currently available.

## Conclusion

Three dietary patterns, one healthy and two unhealthy, were identified in the French-speaking Swiss population. Several associated modifiable behaviours were identified, and this information allows targeting of the most vulnerable groups in the context of public health interventions.

## Additional file

Additional file 1:Table S1. Food groups used to derive dietary patterns, CoLaus study, 2009–2012, Lausanne, Switzerland. **Table S2.** Comparison between excluded and included participants, CoLaus study, 2009–2012, Lausanne, Switzerland. **Table S3.** Pearson correlation coefficients and 95% confidence intervals between dietary patterns scores and daily intakes of selected macro- and micronutrients, 4372 participants of the CoLaus study, 2009–2012, Lausanne, Switzerland. **Table S4.** Multivariable analysis of the associations between personal and behavioural factors and dietary patterns scores, CoLaus study, 2009–2012, Lausanne, Switzerland. **Table S5.** Multivariable analysis of the associations between personal and behavioural factors and being in the highest quintile of the three dietary patterns identified, CoLaus study, 2009–2012, Lausanne, Switzerland. Participants without data for sedentary status (*N* = 436) excluded. **Table S6.** Multivariable analysis of the associations between personal and behavioural factors and dietary patterns scores, CoLaus study, 2009–2012, Lausanne, Switzerland. Participants without data for sedentary status (*N* = 436) excluded. **Table S7.** Multivariable analysis of the associations between personal and behavioural factors and being in the highest quintile of the three dietary patterns identified, CoLaus study, 2009–2012, Lausanne, Switzerland. Participants with extreme energy intakes (< 850 or > 4500 kcal/day, *N* = 162) excluded. **Table S8.** Multivariable analysis of the associations between personal and behavioural factors and dietary patterns scores, CoLaus study, 2009–2012, Lausanne, Switzerland. Participants with extreme energy intakes (< 850 or > 4500 kcal/day, *N* = 162) excluded. **Table S9.** Multivariable analysis of the associations between personal and behavioural factors and being in the highest quintile of the three dietary patterns identified, CoLaus study, 2009–2012, Lausanne, Switzerland. Participants with extreme energy intakes (< 850 or > 4500 kcal/day, *N* = 162) or without data for sedentary status (*N* = 411) excluded. **Table S10.** Multivariable analysis of the associations between personal and behavioural factors and dietary patterns scores, CoLaus study, 2009–2012, Lausanne, Switzerland. Participants with extreme energy intakes (< 850 or > 4500 kcal/day, N = 162) or without data for sedentary status (*N *= 411) excluded. (DOCX 50 kb)

## Abbreviations

BMI: Body mass index
CI: Confidence interval
FFQ: Food frequency questionnaire
KMO: Kaiser-Meyer-Olkin
PCA: Principal components analysis
PRR: Prevalence rate ratio
SES: Socioeconomic status
USA: United States of America

## References

[1] Martinez-Gonzalez MA, Zazpe I, Razquin C, Sanchez-Tainta A, Corella D, Salas-Salvado J, Toledo E, Ros E, Munoz MA, Recondo J (2015). Empirically-derived food patterns and the risk of total mortality and cardiovascular events in the PREDIMED study. Clin Nutr.

[2] Hu FB (2002). Dietary pattern analysis: a new direction in nutritional epidemiology. Curr Opin Lipidol.

[3] Newby PK, Tucker KL (2004). Empirically derived eating patterns using factor or cluster analysis: a review. Nutr Rev.

[4] Jinlin F, Binyou W, Terry C (2007). A new approach to the study of diet and risk of type 2 diabetes. J Postgrad Med.

[5] Tapsell LC, Neale EP, Satija A, Hu FB (2016). Foods, nutrients, and dietary patterns: interconnections and implications for dietary guidelines. Adv Nutr.

[6] Schwerin HS, Stanton JL, Smith JL, Riley AM, Brett BE (1982). Food, eating habits, and health: a further examination of the relationship between food eating patterns and nutritional health. Am J Clin Nutr.

[7] Arruda SP, da Silva AA, Kac G, Goldani MZ, Bettiol H, Barbieri MA (2014). Socioeconomic and demographic factors are associated with dietary patterns in a cohort of young Brazilian adults. BMC Public Health.

[8] Kesse-Guyot E, Bertrais S, Péneau S, Estaquio C, Dauchet L, Vergnaud AC, Czernichow S, Galan P, Hercberg S, Bellisle F (2009). Dietary patterns and their sociodemographic and behavioural correlates in French middle-aged adults from the SU.VI.MAX cohort. Eur J Clin Nutr.

[9] Tucker KL (2010). Dietary patterns, approaches, and multicultural perspective. Appl Physiol Nutr Metab.

[10] de Abreu D, Guessous I, Gaspoz JM, Marques-Vidal P (2014). Compliance with the Swiss Society for Nutrition's dietary recommendations in the population of Geneva, Switzerland: a 10-year trend study (1999-2009). J Acad Nutr Diet.

[11] de Abreu D, Guessous I, Vaucher J, Preisig M, Waeber G, Vollenweider P, Marques-Vidal P (2013). Low compliance with dietary recommendations for food intake among adults. Clin Nutr.

[12] Marques-Vidal P, Gaspoz JM, Theler JM, Guessous I (2017). Twenty-year trends in dietary patterns in French-speaking Switzerland: toward healthier eating. Am J Clin Nutr.

[13] Firmann M, Mayor V, Vidal PM, Bochud M, Pecoud A, Hayoz D, Paccaud F, Preisig M, Song KS, Yuan X (2008). The CoLaus study: a population-based study to investigate the epidemiology and genetic determinants of cardiovascular risk factors and metabolic syndrome. BMC Cardiovasc Disord.

[14] Marques-Vidal P, Waeber G, Vollenweider P, Bochud M, Stringhini S, Guessous I (2015). Sociodemographic and Behavioural determinants of a healthy diet in Switzerland. Ann Nutr Metab.

[15] Bernstein M, Sloutskis D, Kumanyika S, Sparti A, Schutz Y, Morabia A (1998). Data-based approach for developing a physical activity frequency questionnaire. Am J Epidemiol.

[16] Bernstein MS, Morabia A, Sloutskis D (1999). Definition and prevalence of sedentarism in an urban population. Am J Public Health.

[17] Guessous I, Gaspoz JM, Theler JM, Kayser B (2014). Eleven-year physical activity trends in a Swiss urban area. Prev Med.

[18] Bernstein L, Huot I, Morabia A (1995). Amélioration des performances d'un questionnaire alimentaire semi-quantitatif comparé à un rappel des 24 heures. Santé Publique.

[19] Beer-Borst S, Costanza MC, Pechère-Bertschi A, Morabia A (2009). Twelve-year trends and correlates of dietary salt intakes for the general adult population of Geneva Switzerland. Eur J Clin Nutr.

[20] French food composition table [https://pro.anses.fr/tableciqual/index.htm].

[21] Bernstein M, Morabia A, Costanza MC, Landis JR, Ross A, Flandre P, Luong BL, Kumanyika S, Sorenson A, Localio R (1994). Nutritional balance of the diet of the adult residents of Geneva. Sozial und Praventivmedizin.

[22] Fernandez-Alvira JM, Bammann K, Pala V, Krogh V, Barba G, Eiben G, Hebestreit A, Veidebaum T, Reisch L, Tornaritis M (2014). Country-specific dietary patterns and associations with socioeconomic status in European children: the IDEFICS study. Eur J Clin Nutr.

[23] Esmaillzadeh A, Azadbakht L (2008). Major dietary patterns in relation to general obesity and central adiposity among Iranian women. J Nutr.

[24] Markussen MS, Veierod MB, Kristiansen AL, Ursin G, Andersen LF (2016). Dietary patterns of women aged 50-69 years and associations with nutrient intake, sociodemographic factors and key risk factors for non-communicable diseases. Public Health Nutr.

[25] Dziuban CD, Shirkley EC (1974). When is a correlation matrix appropriate for factor analysis? Some decision rules. Psychol Bull.

[26] Barros AJ, Hirakata VN (2003). Alternatives for logistic regression in cross-sectional studies: an empirical comparison of models that directly estimate the prevalence ratio. BMC Med Res Methodol.

[27] Iqbal R, Ajayan K, Bharathi AV, Zhang X, Islam S, Soman CR, Merchant AT (2009). Refinement and validation of an FFQ developed to estimate macro- and micronutrient intakes in a south Indian population. Public Health Nutr.

[28] Healthy diet [(https://cancer-code-europe.iarc.fr/index.php/en/ecac-12-ways/diet-recommendation/39-healthy-diet].

[29] Mattei J, Noel SE, Tucker KL (2011). A meat, processed meat, and French fries dietary pattern is associated with high allostatic load in Puerto Rican older adults. J Am Diet Assoc.

[30] Berg CM, Lappas G, Strandhagen E, Wolk A, Toren K, Rosengren A, Aires N, Thelle DS, Lissner L (2008). Food patterns and cardiovascular disease risk factors: the Swedish INTERGENE research program. Am J Clin Nutr.

[31] Centritto F, Iacoviello L, di Giuseppe R, De Curtis A, Costanzo S, Zito F, Grioni S, Sieri S, Donati MB, de Gaetano G (2009). Dietary patterns, cardiovascular risk factors and C-reactive protein in a healthy Italian population. Nutr Metab Cardiovasc Dis.

[32] Cunha DB, de Almeida RM, Sichieri R, Pereira RA (2010). Association of dietary patterns with BMI and waist circumference in a low-income neighbourhood in Brazil. Br J Nutr.

[33] Knudsen VK, Matthiessen J, Biltoft-Jensen A, Sorensen MR, Groth MV, Trolle E, Christensen T, Fagt S (2014). Identifying dietary patterns and associated health-related lifestyle factors in the adult Danish population. Eur J Clin Nutr.

[34] Fung TT, Schulze M, Manson JE, Willett WC, Hu FB (2004). Dietary patterns, meat intake, and the risk of type 2 diabetes in women. Arch Intern Med.

[35] Oliveira A, Rodríguez-Artalejo F, Gaio R, Santos AC, Ramos E, Lopes C (2011). Major habitual dietary patterns are associated with acute myocardial infarction and cardiovascular risk markers in a southern European population. J Am Diet Assoc.

[36] Marques-Vidal P, Bovet P, Paccaud F, Chiolero A (2010). Changes of overweight and obesity in the adult Swiss population according to educational level, from 1992 to 2007. BMC Public Health.

[37] Imoscopi A, Inelmen EM, Sergi G, Miotto F, Manzato E (2012). Taste loss in the elderly: epidemiology, causes and consequences. Aging Clin Exp Res.

[38] Drewnowski A (2010). The cost of US foods as related to their nutritive value. Am J Clin Nutr.

[39] Marques-Vidal P, Ravasco P, Dias CM, Camilo ME (2006). Trends of food intake in Portugal, 1987-1999: results from the National Health Surveys. Eur J Clin Nutr.

[40] Northstone K, Emmett PM (2010). Dietary patterns of men in ALSPAC: associations with socio-demographic and lifestyle characteristics, nutrient intake and comparison with women's dietary patterns. Eur J Clin Nutr.

[41] Yamauchi Y, Endo S, Yoshimura I (2002). A new whole-mouth gustatory test procedure. II. Effects of aging, gender and smoking. Acta Otolaryngol Suppl.

[42] Vennemann MM, Hummel T, Berger K (2008). The association between smoking and smell and taste impairment in the general population. J Neurol.

[43] Mesas AE, Leon-Munoz LM, Guallar-Castillon P, Graciani A, Gutierrez-Fisac JL, Lopez-Garcia E, Aguilera MT, Banegas JR, Rodriguez-Artalejo F (2012). Obesity-related eating behaviours in the adult population of Spain, 2008-2010. Obes Rev.

[44] Miles A, Rapoport L, Wardle J, Afuape T, Duman M (2001). Using the mass-media to target obesity: an analysis of the characteristics and reported behaviour change of participants in the BBC's 'Fighting fat, fighting Fit' campaign. Health Educ Res.

[45] Nouvelle définition statistique des agglomérations et des villes 2012 [https://www.bfs.admin.ch/bfs/fr/home/statistiques/themes-transversaux/analyses-spatiales.assetdetail.38622.html].

[46] Smith AD, Emmett PM, Newby PK, Northstone K (2013). Dietary patterns obtained through principal components analysis: the effect of input variable quantification. Br J Nutr.

