# Supplementary material for: Socio-demographic and lifestyle determinants of dietary patterns in French-speaking Switzerland, 2009–2012
Source: BMC Public Health. 2018 Jan 12;18:131. doi: 10.1186/s12889-018-5045-1 (PMC5766995; doi:10.1186/s12889-018-5045-1)
Supplement: Additional file 1:Table S1. — Food groups used to derive dietary patterns, CoLaus study, 2009–2012, Lausanne, Switzerland. Table S2. Comparison between excluded and included participants, CoLaus study, 2009–2012, Lausanne, Switzerland. Table S3. Pearson correlation coefficients and 95% confidence intervals between dietary patterns scores and daily intakes of selected macro- and micronutrients, 4372 participants of the CoLaus study, 2009–2012, Lausanne, Switzerland. Table S4. Multivariable analysis of the associations between personal and behavioural factors and dietary patterns scores, CoLaus study, 2009–2012, Lausanne, Switzerland. Table S5. Multivariable analysis of the associations between personal and behavioural factors and being in the highest quintile of the three dietary patterns identified, CoLaus study, 2009–2012, Lausanne, Switzerland. Participants without data for sedentary status (N = 436) excluded. Table S6. Multivariable analysis of the associations between personal and behavioural factors and dietary patterns scores, CoLaus study, 2009–2012, Lausanne, Switzerland. Participants without data for sedentary status (N = 436) excluded. Table S7. Multivariable analysis of the associations between personal and behavioural factors and being in the highest quintile of the three dietary patterns identified, CoLaus study, 2009–2012, Lausanne, Switzerland. Participants with extreme energy intakes (< 850 or > 4500 kcal/day, N = 162) excluded. Table S8. Multivariable analysis of the associations between personal and behavioural factors and dietary patterns scores, CoLaus study, 2009–2012, Lausanne, Switzerland. Participants with extreme energy intakes (< 850 or > 4500 kcal/day, N = 162) excluded. Table S9. Multivariable analysis of the associations between personal and behavioural factors and being in the highest quintile of the three dietary patterns identified, CoLaus study, 2009–2012, Lausanne, Switzerland. Participants with extreme energy intakes (< 850 or > 4500 kcal/day, N = 162) or witho [file 12889_2018_5045_MOESM1_ESM.docx]

**Additional file**

**Additional file 1: Table S1**. Food groups used to derive dietary patterns, CoLaus study, 2009-2012, Lausanne, Switzerland.

| **Food group** | **Examples** |
| --- | --- |
| Full fat or semi-skimmed dairy products | Milk, yogurt |
| Low fat dairy products | Cottage cheese, 0% fat; yogurt, light |
| Whole milk cheese | Emmental, Gruyère, fondue, feta, tomme, parmesan |
| White bread |  |
| Wholemeal bread |  |
| Breakfast cereals | Puffed rice, corn flakes, muesli, porridge |
| Toasts, crackers |  |
| Red meat | Beef, horsemeat, veal, spareribs, lamb chops |
| Poultry | Poultry with or without skin |
| Processed meat | Sausage, salami, cured ham, *cervelas*, pâté, terrine |
| Liver | Liver (veal, pork, poultry) |
| Oily fish | Salmon (fresh or smoked) |
| Canned or fried fish | Tuna in oil |
| Lean fish & seafood | Cod, pollock, trout, shrimp |
| Vegetables | Spinach, green beans, tomatoes, broccoli, carrots, veg. soup |
| Boiled potatoes |  |
| French fries |  |
| Sauces (any) | Mayonnaise, vinaigrette, tomato |
| Cafeteria foods | Ravioli, tortellini, cannelloni, pizza, quiche |
| Starchy foods | Rice, pasta, semolina |
| Eggs |  |
| Tofu |  |
| Fresh fruit | Banana, pear, apple, plum, grapes, berries, orange, tangerine, melon, apricot, fresh fruit juice |
| Canned fruit |  |
| Low-cal fat products | Low-fat margarine, low-fat cream |
| Hard fats | Butter, margarine |
| Olive oil |  |
| Other vegetable oils | Sunflower seed, peanut |
| Bakery | Croissant, chocolate bread, cream cake, English cake, biscuits, cookies, fruit pie |
| Chocolate | Chocolate, jam, honey, ice-cream, sugar |
| Sugar substitutes | Aspartame |
| Vitamin supplements | Single or multivitamins |
| Other supplements | Fibre, bran, garlic pills |
| Sodas | Sodas, bottled fruit juice |
| Tea & coffee |  |
| Water |  |
| Alcoholic drinks | Beer, wine, champagne, spirits, aperitifs |

**Additional file 1: Table S2**. Comparison between excluded and included participants, CoLaus study, 2009-2012, Lausanne, Switzerland.

|  | **Included** | **Excluded** | **p-value** |
| --- | --- | --- | --- |
| Sample size | 4,372 | 692 |  |
| Gender (%) |  |  |  |
| Woman | 2359 (54.0) | 348 (50.3) | 0.07 |
| Man | 2013 (46.0) | 344 (49.7) |  |
| Age (years) | 57.3 ± 10.3 | 60.4 ± 11.5 | <0.001 |
| Age groups (%) |  |  | 0.002 |
| 40-49 | 1279 (29.3) | 152 (23.4) |  |
| 50-59 | 1347 (30.8) | 195 (30.1) |  |
| 60-69 | 1163 (26.6) | 188 (29.0) |  |
| 70-79 | 583 (13.3) | 114 (17.6) |  |
| Country of birth (%) |  |  | <0.001 |
| Switzerland | 2844 (65.1) | 340 (49.1) |  |
| France | 290 (6.6) | 28 (4.1) |  |
| Italy | 212 (4.9) | 66 (9.5) |  |
| Portugal | 200 (4.6) | 53 (7.7) |  |
| Spain | 134 (3.1) | 44 (6.4) |  |
| Other | 692 (15.8) | 161 (23.3) |  |
| Smoking status (%) |  |  | <0.001 |
| Never | 1807 (41.3) | 228 (35.9) |  |
| Former | 1662 (38.0) | 221 (34.8) |  |
| Current | 903 (20.7) | 186 (29.3) |  |
| Educational level (%) |  |  | <0.001 |
| University | 982 (22.5) | 97 (14.0) |  |
| High school/college | 1166 (26.7) | 140 (20.2) |  |
| Apprenticeship | 1560 (35.7) | 236 (34.1) |  |
| Basic | 664 (15.2) | 219 (31.7) |  |
| On a diet (%) | 1379 (31.5) | 195 (28.2) | 0.08 |
| Sedentary (%) | 2238 (56.9) | 167 (68.2) | 0.001 |
| BMI (kg/m^2^) | 26.1 ± 4.5 | 27.0 ± 5.1 | <0.001 |
| BMI categories (%) |  |  | <0.001 |
| Normal | 1979 (45.3) | 266 (38.4) |  |
| Overweight | 1683 (38.5) | 277 (40.0) |  |
| Obese | 710 (16.2) | 149 (21.5) |  |

Results are expressed as mean ± standard deviation or as number of participants and (column %). BMI, body mass index. Statistical analysis using chi-square or student’s t-test.

**Additional file 1: Table S3**. Pearson correlation coefficients and 95% confidence intervals between dietary patterns scores and daily intakes of selected macro- and micronutrients, 4,372 participants of the CoLaus study, 2009-2012, Lausanne, Switzerland.

|  | **Meat & fries** | **Fruits & vegetables** | **Fatty & sugary** | **p-value ^1^** | **p-value ^2^** | **p-value ^3^** |
| --- | --- | --- | --- | --- | --- | --- |
| Total energy intake | 0.449 (0.425 ; 0.472) | 0.430 (0.406 ; 0.454) | 0.678 (0.661 ; 0.694) | 0.259 | <0.001 | <0.001 |
| Macronutrients (as % TEI) |  |  |  |  |  |  |
| Total protein | 0.268 (0.240 ; 0.295) | 0.083 (0.053 ; 0.112) | -0.377 (-0.402 ; -0.351) | <0.001 | 0.100 | 0.002 |
| Vegetable protein | -0.155 (-0.183 ; -0.125) | 0.175 (0.146 ; 0.203) | -0.058 (-0.088 ; -0.028) | <0.001 | <0.001 | <0.001 |
| Animal protein | 0.289 (0.262 ; 0.316) | 0.017 (-0.013 ; 0.047) | -0.317 (-0.343 ; -0.290) | <0.001 | <0.001 | <0.001 |
| Total carbohydrates | -0.244 (-0.272 ; -0.216) | 0.095 (0.066 ; 0.125) | 0.096 (0.067 ; 0.126) | <0.001 | 0.959 | <0.001 |
| Mono and disaccharides | -0.243 (-0.270 ; -0.215) | 0.166 (0.137 ; 0.195) | 0.009 (-0.021 ; 0.039) | <0.001 | <0.001 | <0.001 |
| Polysaccharides | -0.020 (-0.050 ; 0.009) | -0.066 (-0.095 ; -0.036) | 0.099 (0.069 ; 0.128) | 0.017 | <0.001 | <0.001 |
| Total fat | 0.140 (0.111 ; 0.169) | 0.067 (0.038 ; 0.097) | 0.102 (0.073 ; 0.131) | <0.001 | 0.082 | 0.046 |
| SFA | 0.070 (0.041 ; 0.100) | -0.187 (-0.215 ; -0.158) | 0.265 (0.237 ; 0.292) | <0.001 | <0.001 | <0.001 |
| MUFA | 0.137 (0.108 ; 0.166) | 0.156 (0.127 ; 0.185) | -0.008 (-0.038 ; 0.021) | 0.315 | <0.001 | <0.001 |
| PUFA | 0.140 (0.110 ; 0.168) | 0.204 (0.176 ; 0.233) | -0.054 (-0.084 ; -0.024) | <0.001 | <0.001 | <0.001 |
| Micronutrients (per day) |  |  |  |  |  |  |
| Dietary fibre (g) | 0.106 (0.077 ; 0.135) | 0.628 (0.610 ; 0.646) | 0.277 (0.250 ; 0.304) | <0.001 | <0.001 | <0.001 |
| Cholesterol (mg) | 0.555 (0.534 ; 0.575) | 0.325 (0.298 ; 0.351) | 0.458 (0.434 ; 0.481) | <0.001 | <0.001 | <0.001 |
| Alcohol (g) | 0.140 (0.111 ; 0.169) | -0.192 (-0.220 ; -0.163) | 0.114 (0.085 ; 0.143) | <0.001 | <0.001 | 0.176 |
| Calcium (mg) | 0.090 (0.060 ; 0.119) | 0.327 (0.300 ; 0.353) | 0.380 (0.354 ; 0.405) | <0.001 | 0.004 | <0.001 |
| Iron (mg) | 0.558 (0.537 ; 0.578) | 0.548 (0.526 ; 0.568) | 0.435 (0.411 ; 0.459) | 0.460 | <0.001 | <0.001 |
| Retinol (μg) | 0.577 (0.557 ; 0.597) | 0.248 (0.220 ; 0.276) | 0.160 (0.131 ; 0.189) | <0.001 | <0.001 | <0.001 |
| Carotene (μg) | 0.158 (0.129 ; 0.187) | 0.533 (0.511 ; 0.554) | 0.115 (0.085 ; 0.144) | <0.001 | <0.001 | 0.023 |
| Vitamin D (μg) | 0.335 (0.308 ; 0.361) | 0.451 (0.427 ; 0.474) | 0.059 (0.029 ; 0.088) | <0.001 | <0.001 | <0.001 |

SFA, saturated fatty acids; MUFA, monounsaturated fatty acids; PUFA, polyunsaturated fatty acids; TEI, total energy intake. Pairwise comparisons of correlation coefficients were performed using Steiger’s method and the **corcor** command of Stata. ^1^, “Meat & fries” vs. “Fruits & Vegetables”; ^2^, “Fruits & Vegetables” vs. “Fatty & sugary”; ^3^, “Meat & fries” vs. “Fatty & sugary”.

**Additional file 1: Table S4**. Multivariable analysis of the associations between personal and behavioural factors and dietary patterns scores, CoLaus study, 2009-2012, Lausanne, Switzerland.

|  | **Meat & fries** | **Fruits & vegetables** | **Fatty & sugary** |
| --- | --- | --- | --- |
| Gender |  |  |  |
| Woman | -0.229 ± 0.036 | 0.379 ± 0.032 | -0.148 ± 0.030 |
| Man | 0.268 ± 0.039 | -0.444 ± 0.035 | 0.173 ± 0.032 |
| p-value | <0.001 | <0.001 | <0.001 |
| Age group |  |  |  |
| 40-49 | 0.143 ± 0.048 | -0.136 ± 0.044 | -0.007 ± 0.040 |
| 50-59 | 0.050 ± 0.046 | 0.026 ± 0.042 | -0.050 ± 0.039 |
| 60-69 | -0.104 ± 0.050 | 0.092 ± 0.046 | -0.060 ± 0.042 |
| 70-79 | -0.221 ± 0.072 | 0.054 ± 0.065 | 0.250 ± 0.060 |
| *p-value for trend* | <0.001 | 0.011 | 0.001 |
| Country of birth |  |  |  |
| Switzerland | -0.108 ± 0.032 | -0.113 ± 0.029 | 0.002 ± 0.027 |
| France | 0.185 ± 0.100 | 0.048 ± 0.091 | 0.174 ± 0.083 |
| Italy | -0.096 ± 0.118 | 0.134 ± 0.107 | -0.015 ± 0.098 |
| Portugal | 0.580 ± 0.131 | 0.605 ± 0.119 | 0.009 ± 0.109 |
| Spain | 0.426 ± 0.148 | 0.308 ± 0.134 | -0.259 ± 0.124 |
| Other | 0.144 ± 0.066 | 0.168 ± 0.060 | -0.028 ± 0.055 |
| p-value | <0.001 | <0.001 | 0.106 |
| Education |  |  |  |
| University | -0.070 ± 0.056 | 0.275 ± 0.051 | -0.135 ± 0.047 |
| High school | -0.060 ± 0.050 | 0.082 ± 0.045 | -0.001 ± 0.042 |
| Apprenticeship | 0.030 ± 0.044 | -0.137 ± 0.040 | 0.069 ± 0.037 |
| Primary | 0.138 ± 0.073 | -0.229 ± 0.066 | 0.040 ± 0.061 |
| *p-value for trend* | 0.016 | <0.001 | 0.016 |
| Smoking status |  |  |  |
| Never | 0.043 ± 0.040 | 0.101 ± 0.036 | 0.007 ± 0.033 |
| Former | -0.083 ± 0.042 | 0.078 ± 0.038 | -0.016 ± 0.035 |
| Current | 0.067 ± 0.057 | -0.346 ± 0.052 | 0.016 ± 0.047 |
| *p-value for trend* | 0.736 | <0.001 | 0.885 |
| On a diet |  |  |  |
| No | 0.032 ± 0.031 | -0.127 ± 0.028 | 0.148 ± 0.026 |
| Yes | -0.070 ± 0.046 | 0.276 ± 0.042 | -0.320 ± 0.039 |
| p-value | <0.001 | <0.001 | <0.001 |
| BMI categories |  |  |  |
| Normal | -0.139 ± 0.039 | 0.074 ± 0.035 | 0.100 ± 0.033 |
| Overweight | 0.072 ± 0.042 | 0.003 ± 0.038 | -0.037 ± 0.035 |
| Obese | 0.216 ± 0.065 | -0.212 ± 0.059 | -0.190 ± 0.054 |
| *p-value for trend* | <0.001 | <0.001 | <0.001 |

Analysis conducted on 4,372 participants. Results are expressed as adjusted mean ± standard error. Statistical analysis performed by analysis of variance simultaneously adjusting for all the variables listed in the table.

**Additional file 1: Table S5**. Multivariable analysis of the associations between personal and behavioural factors and being in the highest quintile of the three dietary patterns identified, CoLaus study, 2009-2012, Lausanne, Switzerland. Participants without data for sedentary status (N=436) excluded.

|  | **Meat & fries** | **Fruits & vegetables** | **Fatty & sugary** |
| --- | --- | --- | --- |
| Gender |  |  |  |
| Woman | 1 (ref.) | 1 (ref.) | 1 (ref.) |
| Man | 1.92 (1.65 - 2.24) | 0.48 (0.41 - 0.57) | 1.40 (1.21 - 1.62) |
| p-value | <0.001 | <0.001 | <0.001 |
| Age group |  |  |  |
| 40-49 | 1 (ref.) | 1 (ref.) | 1 (ref.) |
| 50-59 | 0.92 (0.78 - 1.09) | 1.18 (0.98 - 1.42) | 1.05 (0.88 - 1.26) |
| 60-69 | 0.69 (0.56 - 0.85) | 1.46 (1.20 - 1.77) | 0.96 (0.79 - 1.17) |
| 70-79 | 0.67 (0.51 - 0.88) | 1.23 (0.96 - 1.59) | 1.29 (1.03 - 1.63) |
| *p-value for trend* | <0.001 | 0.039 | 0.068 |
| Country of birth |  |  |  |
| Switzerland | 1 (ref.) | 1 (ref.) | 1 (ref.) |
| France | 1.54 (1.18 - 2.02) ** | 1.07 (0.81 - 1.41) | 1.37 (1.06 - 1.77) * |
| Italy | 0.86 (0.59 - 1.25) | 1.22 (0.86 - 1.73) | 1.08 (0.78 - 1.49) |
| Portugal | 2.07 (1.56 - 2.76) *** | 2.10 (1.51 - 2.92) *** | 0.90 (0.60 - 1.34) |
| Spain | 2.01 (1.46 - 2.77) *** | 1.39 (0.94 - 2.06) | 0.85 (0.54 - 1.35) |
| Other | 1.50 (1.23 - 1.83) *** | 1.14 (0.94 - 1.38) | 1.12 (0.91 - 1.37) |
| Education |  |  |  |
| University | 1 (ref.) | 1 (ref.) | 1 (ref.) |
| High school | 1.00 (0.81 - 1.22) | 0.82 (0.68 - 0.99) | 1.08 (0.88 - 1.32) |
| Apprenticeship | 1.08 (0.89 - 1.33) | 0.63 (0.51 - 0.76) | 1.17 (0.96 - 1.42) |
| Primary | 1.26 (0.98 - 1.63) | 0.65 (0.51 - 0.85) | 1.16 (0.89 - 1.51) |
| *p-value for trend* | 0.052 | <0.001 | 0.213 |
| Smoking status |  |  |  |
| Never | 1 (ref.) | 1 (ref.) | 1 (ref.) |
| Former | 0.94 (0.80 - 1.11) | 1.08 (0.93 - 1.26) | 1.10 (0.94 - 1.29) |
| Current | 1.16 (0.97 - 1.39) | 0.68 (0.55 - 0.85) | 1.17 (0.97 - 1.40) |
| *p-value for trend* | 0.108 | <0.001 | 0.106 |
| On a diet |  |  |  |
| No | 1 (ref.) | 1 (ref.) | 1 (ref.) |
| Yes | 0.84 (0.71 - 0.99) | 1.49 (1.29 - 1.73) | 0.68 (0.57 - 0.80) |
| p-value | 0.03 | <0.001 | <0.001 |
| BMI categories |  |  |  |
| Normal | 1 (ref.) | 1 (ref.) | 1 (ref.) |
| Overweight | 1.19 (1.02 - 1.40) | 0.93 (0.80 - 1.09) | 0.90 (0.77 - 1.05) |
| Obese | 1.24 (1.00 - 1.53) | 0.86 (0.69 - 1.07) | 0.71 (0.57 - 0.90) |
| *p-value for trend* | 0.046 | 0.188 | 0.004 |
| Sedentary |  |  |  |
| No | 1 (ref.) | 1 (ref.) | 1 (ref.) |
| Yes | 1.20 (1.04 - 1.39) | 0.79 (0.69 - 0.91) | 1.11 (0.96 - 1.28) |
| p-value | 0.010 | <0.001 | 0.155 |

Analysis conducted on 3,936 participants. Results are expressed as prevalence rate ratios and (95% confidence interval) of being in the last quintile relative to the other four. Statistical analysis performed using Poisson regression adjusting for the variables listed in the tables. All variables were simultaneously included in the model. For country of birth, significant associations are indicated as follows: *, p<0.05; **, p<0.01; ***, p<0.001.

**Additional file1: Table S6**. Multivariable analysis of the associations between personal and behavioural factors and dietary patterns scores, CoLaus study, 2009-2012, Lausanne, Switzerland. Participants without data for sedentary status (N=436) excluded.

|  | **Meat & fries** | **Fruits & vegetables** | **Fatty & sugary** |
| --- | --- | --- | --- |
| Gender |  |  |  |
| Woman | -0.241 ± 0.038 | 0.401 ± 0.034 | -0.133 ± 0.031 |
| Man | 0.276 ± 0.042 | -0.424 ± 0.037 | 0.206 ± 0.034 |
| p-value | <0.001 | <0.001 | <0.001 |
| Age group |  |  |  |
| 40-49 | 0.138 ± 0.052 | -0.142 ± 0.046 | 0.001 ± 0.042 |
| 50-59 | 0.052 ± 0.049 | 0.045 ± 0.044 | -0.022 ± 0.040 |
| 60-69 | -0.114 ± 0.055 | 0.142 ± 0.048 | -0.018 ± 0.044 |
| 70-79 | -0.252 ± 0.079 | 0.116 ± 0.070 | 0.261 ± 0.064 |
| *p-value for trend* | <0.001 | 0.001 | 0.001 |
| Country of birth |  |  |  |
| Switzerland | -0.104 ± 0.035 | -0.080 ± 0.031 | 0.030 ± 0.028 |
| France | 0.183 ± 0.108 | 0.057 ± 0.096 | 0.199 ± 0.088 |
| Italy | -0.150 ± 0.129 | 0.183 ± 0.114 | -0.003 ± 0.105 |
| Portugal | 0.540 ± 0.144 | 0.624 ± 0.127 | -0.013 ± 0.117 |
| Spain | 0.400 ± 0.161 | 0.192 ± 0.142 | -0.285 ± 0.130 |
| Other | 0.143 ± 0.071 | 0.189 ± 0.063 | -0.005 ± 0.058 |
| p-value | <0.001 | <0.001 | 0.080 |
| Education |  |  |  |
| University | -0.075 ± 0.060 | 0.274 ± 0.053 | -0.110 ± 0.049 |
| High school | -0.062 ± 0.054 | 0.096 ± 0.047 | 0.015 ± 0.044 |
| Apprenticeship | 0.019 ± 0.047 | -0.113 ± 0.042 | 0.098 ± 0.038 |
| Primary | 0.158 ± 0.080 | -0.175 ± 0.071 | 0.059 ± 0.065 |
| *p-value for trend* | 0.016 | <0.001 | 0.026 |
| Smoking status |  |  |  |
| Never | 0.036 ± 0.043 | 0.104 ± 0.038 | 0.027 ± 0.035 |
| Former | -0.083 ± 0.045 | 0.095 ± 0.040 | 0.001 ± 0.037 |
| Current | 0.059 ± 0.062 | -0.276 ± 0.055 | 0.051 ± 0.050 |
| *p-value for trend* | 0.761 | <0.001 | 0.694 |
| On a diet |  |  |  |
| No | 0.030 ± 0.034 | -0.112 ± 0.030 | 0.165 ± 0.027 |
| Yes | -0.080 ± 0.050 | 0.322 ± 0.045 | -0.294 ± 0.041 |
| p-value | <0.001 | <0.001 | <0.001 |
| BMI categories |  |  |  |
| Normal | -0.123 ± 0.042 | 0.082 ± 0.037 | 0.129 ± 0.034 |
| Overweight | 0.075 ± 0.045 | 0.025 ± 0.040 | -0.013 ± 0.036 |
| Obese | 0.144 ± 0.071 | -0.154 ± 0.063 | -0.202 ± 0.058 |
| *p-value for trend* | <0.002 | 0.002 | <0.001 |
| Sedentary |  |  |  |
| No | -0.115 ± 0.043 | 0.169 ± 0.038 | -0.042 ± 0.035 |
| Yes | 0.079 ± 0.037 | -0.087 ± 0.033 | 0.071 ± 0.030 |
| p-value | <0.001 | <0.001 | 0.015 |

Analysis conducted on 3,936 participants. Results are expressed as adjusted mean ± standard error. Statistical analysis performed by analysis of variance simultaneously adjusting for all the variables listed in the table.

**Additional file 1: Table S7**. Multivariable analysis of the associations between personal and behavioural factors and being in the highest quintile of the three dietary patterns identified, CoLaus study, 2009-2012, Lausanne, Switzerland. Participants with extreme energy intakes (<850 or >4500 kcal/day, N=162) excluded.

|  | **Meat & fries** | **Fruits & vegetables** | **Fatty & sugary** |
| --- | --- | --- | --- |
| Gender |  |  |  |
| Woman | 1 (ref.) | 1 (ref.) | 1 (ref.) |
| Man | 1.84 (1.59 - 2.13) | 0.46 (0.40 - 0.54) | 1.30 (1.13 - 1.50) |
| p-value | <0.001 | <0.001 | <0.001 |
| Age group |  |  |  |
| 40-49 | 1 (ref.) | 1 (ref.) | 1 (ref.) |
| 50-59 | 0.90 (0.77 - 1.06) | 1.14 (0.95 - 1.37) | 1.01 (0.85 - 1.20) |
| 60-69 | 0.71 (0.58 - 0.86) | 1.36 (1.13 - 1.64) | 0.92 (0.76 - 1.12) |
| 70-79 | 0.70 (0.55 - 0.91) | 1.16 (0.91 - 1.48) | 1.27 (1.02 - 1.58) |
| *p-value for trend* | 0.001 | 0.111 | 0.077 |
| Country of birth |  |  |  |
| Switzerland | 1 (ref.) | 1 (ref.) | 1 (ref.) |
| France | 1.47 (1.13 - 1.92) ** | 1.00 (0.76 - 1.32) | 1.32 (1.02 - 1.70) * |
| Italy | 0.97 (0.69 - 1.36) | 1.21 (0.87 - 1.69) | 1.05 (0.77 - 1.44) |
| Portugal | 2.00 (1.52 - 2.63) *** | 2.02 (1.47 - 2.79) *** | 0.87 (0.60 - 1.26) |
| Spain | 1.90 (1.39 - 2.60) *** | 1.55 (1.08 - 2.22) | 0.89 (0.58 - 1.36) |
| Other | 1.52 (1.26 - 1.84) *** | 1.13 (0.94 - 1.36) | 1.10 (0.90 - 1.33) |
| Education |  |  |  |
| University | 1 (ref.) | 1 (ref.) | 1 (ref.) |
| High school | 1.01 (0.82 - 1.23) | 0.81 (0.67 - 0.97) | 1.07 (0.88 - 1.30) |
| Apprenticeship | 1.07 (0.88 - 1.30) | 0.60 (0.49 - 0.72) | 1.14 (0.94 - 1.38) |
| Primary | 1.29 (1.02 - 1.65) | 0.63 (0.49 - 0.81) | 1.27 (0.99 - 1.63) |
| *p-value for trend* | 0.031 | <0.001 | 0.047 |
| Smoking status |  |  |  |
| Never | 1 (ref.) | 1 (ref.) | 1 (ref.) |
| Former | 0.94 (0.8 - 1.10) | 1.09 (0.94 - 1.26) | 1.15 (0.98 - 1.34) |
| Current | 1.16 (0.97 - 1.38) | 0.68 (0.55 - 0.84) | 1.22 (1.02 - 1.45) |
| *p-value for trend* | 0.101 | <0.001 | 0.033 |
| On a diet |  |  |  |
| No | 1 (ref.) | 1 (ref.) | 1 (ref.) |
| Yes | 0.86 (0.74 - 1.01) | 1.47 (1.28 - 1.70) | 0.68 (0.58 - 0.80) |
| p-value | 0.060 | <0.001 | <0.001 |
| BMI categories |  |  |  |
| Normal | 1 (ref.) | 1 (ref.) | 1 (ref.) |
| Overweight | 1.21 (1.03 - 1.41) | 0.93 (0.8 - 1.08) | 0.89 (0.77 - 1.04) |
| Obese | 1.41 (1.16 - 1.71) | 0.81 (0.66 - 1.00) | 0.72 (0.57 - 0.89) |
| *p-value for trend* | <0.001 | 0.052 | 0.003 |

Analysis performed on 4,210 participants. Results are expressed as prevalence rate ratios and (95% confidence interval) of being in the last quintile relative to the other four. Statistical analysis performed using Poisson regression adjusting for the variables listed in the tables. All variables were simultaneously included in the model. For country of birth, significant associations are indicated as follows: *, p<0.05; ***, p<0.001.

**Additional file 1: Table S8**. Multivariable analysis of the associations between personal and behavioural factors and dietary patterns scores, CoLaus study, 2009-2012, Lausanne, Switzerland. Participants with extreme energy intakes (<850 or >4500 kcal/day, N=162) excluded.

|  | **Meat & fries** | **Fruits & vegetables** | **Fatty & sugary** |
| --- | --- | --- | --- |
| Gender |  |  |  |
| Woman | -0.236 ± 0.024 | 0.439 ± 0.031 | -0.080 ± 0.029 |
| Man | 0.222 ± 0.025 | -0.451 ± 0.034 | 0.162 ± 0.031 |
| p-value | <0.001 | <0.001 | <0.001 |
| Age group |  |  |  |
| 40-49 | 0.149 ± 0.032 | -0.113 ± 0.042 | 0.038 ± 0.039 |
| 50-59 | 0.009 ± 0.031 | 0.033 ± 0.041 | -0.030 ± 0.037 |
| 60-69 | -0.154 ± 0.033 | 0.125 ± 0.044 | -0.023 ± 0.041 |
| 70-79 | -0.205 ± 0.047 | 0.091 ± 0.063 | 0.281 ± 0.058 |
| *p-value for trend* | <0.001 | 0.004 | 0.001 |
| Country of birth |  |  |  |
| Switzerland | -0.117 ± 0.021 | -0.085 ± 0.028 | 0.040 ± 0.026 |
| France | 0.078 ± 0.067 | -0.028 ± 0.088 | 0.124 ± 0.081 |
| Italy | -0.077 ± 0.078 | 0.165 ± 0.104 | 0.011 ± 0.095 |
| Portugal | 0.605 ± 0.086 | 0.640 ± 0.115 | -0.035 ± 0.106 |
| Spain | 0.468 ± 0.098 | 0.357 ± 0.130 | -0.237 ± 0.120 |
| Other | 0.074 ± 0.044 | 0.197 ± 0.058 | 0.046 ± 0.054 |
| p-value | <0.001 | <0.001 | 0.257 |
| Education |  |  |  |
| University | -0.062 ± 0.037 | 0.303 ± 0.049 | -0.108 ± 0.045 |
| High school | -0.055 ± 0.033 | 0.109 ± 0.044 | 0.035 ± 0.040 |
| Apprenticeship | -0.028 ± 0.029 | -0.143 ± 0.039 | 0.076 ± 0.036 |
| Primary | 0.119 ± 0.049 | -0.169 ± 0.065 | 0.144 ± 0.060 |
| *p-value for trend* | 0.004 | <0.001 | 0.001 |
| Smoking status |  |  |  |
| Never | -0.025 ± 0.026 | 0.097 ± 0.035 | 0.028 ± 0.032 |
| Former | -0.073 ± 0.028 | 0.109 ± 0.037 | 0.019 ± 0.034 |
| Current | 0.085 ± 0.038 | -0.293 ± 0.050 | 0.071 ± 0.046 |
| *p-value for trend* | 0.017 | <0.001 | 0.448 |
| On a diet |  |  |  |
| No | -0.002 ± 0.021 | -0.115 ± 0.027 | 0.167 ± 0.025 |
| Yes | -0.062 ± 0.031 | 0.321 ± 0.041 | -0.259 ± 0.038 |
| p-value | 0.112 | <0.001 | <0.001 |
| BMI categories |  |  |  |
| Normal | -0.130 ± 0.026 | 0.112 ± 0.034 | 0.135 ± 0.032 |
| Overweight | 0.044 ± 0.027 | 0.020 ± 0.037 | 0.011 ± 0.034 |
| Obese | 0.130 ± 0.043 | -0.228 ± 0.057 | -0.199 ± 0.052 |
| *p-value for trend* | <0.001 | <0.001 | <0.001 |

Analysis performed on 4,210 participants. Results are expressed as adjusted mean ± standard error. Statistical analysis performed by analysis of variance simultaneously adjusting for all the variables listed in the table.

**Additional file 1: Table S9**. Multivariable analysis of the associations between personal and behavioural factors and being in the highest quintile of the three dietary patterns identified, CoLaus study, 2009-2012, Lausanne, Switzerland. Participants with extreme energy intakes (<850 or >4500 kcal/day, N=162) or without data for sedentary status (N=411) excluded.

|  | **Meat & fries** | **Fruits & vegetables** | **Fatty & sugary** |
| --- | --- | --- | --- |
| Gender |  |  |  |
| Woman | 1 (ref.) | 1 (ref.) | 1 (ref.) |
| Man | 1.89 (1.62 - 2.20) | 0.46 (0.39 - 0.54) | 1.34 (1.16 - 1.56) |
| p-value | <0.001 | <0.001 | <0.001 |
| Age group |  |  |  |
| 40-49 | 1 (ref.) | 1 (ref.) | 1 (ref.) |
| 50-59 | 0.90 (0.76 - 1.06) | 1.16 (0.96 - 1.40) | 1.02 (0.85 - 1.23) |
| 60-69 | 0.68 (0.55 - 0.84) | 1.45 (1.19 - 1.76) | 0.94 (0.77 - 1.15) |
| 70-79 | 0.66 (0.50 - 0.87) | 1.24 (0.96 - 1.60) | 1.28 (1.02 - 1.62) |
| *p-value for trend* | <0.001 | 0.036 | 0.077 |
| Country of birth |  |  |  |
| Switzerland | 1 (ref.) | 1 (ref.) | 1 (ref.) |
| France | 1.54 (1.17 - 2.03) ** | 0.99 (0.75 - 1.33) | 1.32 (1.01 - 1.73) * |
| Italy | 0.84 (0.57 - 1.24) | 1.25 (0.88 - 1.77) | 1.08 (0.78 - 1.50) |
| Portugal | 2.02 (1.51 - 2.70) *** | 2.08 (1.49 - 2.91) *** | 0.85 (0.57 - 1.28) |
| Spain | 1.99 (1.44 - 2.75) *** | 1.45 (0.98 - 2.15) | 0.83 (0.52 - 1.32) |
| Other | 1.50 (1.23 - 1.83) *** | 1.15 (0.95 - 1.39) | 1.12 (0.91 - 1.37) |
| Education |  |  |  |
| University | 1 (ref.) | 1 (ref.) | 1 (ref.) |
| High school | 1.01 (0.82 - 1.24) | 0.82 (0.68 - 0.99) | 1.09 (0.89 - 1.34) |
| Apprenticeship | 1.08 (0.88 - 1.33) | 0.61 (0.50 - 0.75) | 1.15 (0.95 - 1.41) |
| Primary | 1.30 (1.00 - 1.68) | 0.66 (0.51 - 0.86) | 1.21 (0.92 - 1.58) |
| *p-value for trend* | 0.036 | <0.001 | 0.145 |
| Smoking status |  |  |  |
| Never | 1 (ref.) | 1 (ref.) | 1 (ref.) |
| Former | 0.96 (0.81 - 1.13) | 1.10 (0.94 - 1.27) | 1.12 (0.95 - 1.31) |
| Current | 1.19 (0.99 - 1.43) | 0.72 (0.58 - 0.89) | 1.20 (0.99 - 1.45) |
| *p-value for trend* | 0.064 | 0.003 | 0.058 |
| On a diet |  |  |  |
| No | 1 (ref.) | 1 (ref.) | 1 (ref.) |
| Yes | 0.85 (0.72 - 1.01) | 1.54 (1.33 - 1.78) | 0.69 (0.58 - 0.82) |
| p-value | 0.061 | <0.001 | <0.001 |
| BMI categories |  |  |  |
| Normal | 1 (ref.) | 1 (ref.) | 1 (ref.) |
| Overweight | 1.19 (1.01 - 1.41) | 0.92 (0.78 - 1.07) | 0.91 (0.77 - 1.06) |
| Obese | 1.21 (0.97 - 1.50) | 0.82 (0.65 - 1.02) | 0.69 (0.54 - 0.87) |
| *p-value for trend* | 0.085 | 0.076 | 0.002 |
| Sedentary |  |  |  |
| No | 1 (ref.) | 1 (ref.) | 1 (ref.) |
| Yes | 1.22 (1.05 - 1.41) | 0.79 (0.69 - 0.92) | 1.11 (0.96 - 1.29) |
| p-value | 0.009 | <0.001 | 0.154 |

Analysis performed on 3,799 participants. 25 participants had both extreme energy intakes and lacked data for sedentary status. Results are expressed as prevalence rate ratios and (95% confidence interval) of being in the last quintile relative to the other four. Statistical analysis performed using Poisson regression adjusting for the variables listed in the tables. All variables were simultaneously included in the model. For country of birth, significant associations are indicated as follows: *, p<0.05; ***, p<0.001.

**Additional file 1: Table S10**. Multivariable analysis of the associations between personal and behavioural factors and dietary patterns scores, CoLaus study, 2009-2012, Lausanne, Switzerland. Participants with extreme energy intakes (<850 or >4500 kcal/day, N=162) or without data for sedentary status (N=411) excluded.

|  | **Meat & fries** | **Fruits & vegetables** | **Fatty & sugary** |
| --- | --- | --- | --- |
| Gender |  |  |  |
| Woman | -0.253 ± 0.025 | 0.452 ± 0.033 | -0.073 ± 0.030 |
| Man | 0.228 ± 0.027 | -0.432 ± 0.035 | 0.197 ± 0.033 |
| p-value | <0.001 | <0.001 | <0.001 |
| Age group |  |  |  |
| 40-49 | 0.149 ± 0.033 | -0.119 ± 0.044 | 0.050 ± 0.041 |
| 50-59 | -0.001 ± 0.032 | 0.043 ± 0.042 | -0.010 ± 0.039 |
| 60-69 | -0.175 ± 0.035 | 0.167 ± 0.047 | 0.013 ± 0.043 |
| 70-79 | -0.225 ± 0.051 | 0.154 ± 0.068 | 0.293 ± 0.063 |
| *p-value for trend* | <0.001 | <0.001 | 0.002 |
| Country of birth |  |  |  |
| Switzerland | -0.119 ± 0.022 | -0.058 ± 0.029 | 0.062 ± 0.027 |
| France | 0.108 ± 0.070 | -0.027 ± 0.093 | 0.181 ± 0.086 |
| Italy | -0.131 ± 0.083 | 0.212 ± 0.110 | 0.009 ± 0.102 |
| Portugal | 0.550 ± 0.092 | 0.651 ± 0.122 | -0.071 ± 0.113 |
| Spain | 0.450 ± 0.103 | 0.246 ± 0.137 | -0.255 ± 0.127 |
| Other | 0.063 ± 0.046 | 0.214 ± 0.061 | 0.066 ± 0.056 |
| p-value | <0.001 | <0.001 | 0.111 |
| Education |  |  |  |
| University | -0.073 ± 0.038 | 0.300 ± 0.051 | -0.086 ± 0.047 |
| High school | -0.062 ± 0.034 | 0.124 ± 0.045 | 0.052 ± 0.042 |
| Apprenticeship | -0.039 ± 0.030 | -0.123 ± 0.040 | 0.104 ± 0.037 |
| Primary | 0.128 ± 0.052 | -0.134 ± 0.069 | 0.150 ± 0.064 |
| *p-value for trend* | 0.003 | <0.001 | 0.003 |
| Smoking status |  |  |  |
| Never | -0.037 ± 0.027 | 0.096 ± 0.036 | 0.049 ± 0.034 |
| Former | -0.077 ± 0.029 | 0.120 ± 0.038 | 0.032 ± 0.035 |
| Current | 0.077 ± 0.040 | -0.224 ± 0.053 | 0.098 ± 0.049 |
| *p-value for trend* | 0.018 | <0.001 | 0.408 |
| On a diet |  |  |  |
| No | -0.010 ± 0.021 | -0.105 ± 0.028 | 0.182 ± 0.026 |
| Yes | -0.074 ± 0.032 | 0.365 ± 0.043 | -0.237 ± 0.040 |
| p-value | 0.103 | <0.001 | <0.001 |
| BMI categories |  |  |  |
| Normal | -0.113 ± 0.027 | 0.120 ± 0.036 | 0.161 ± 0.033 |
| Overweight | 0.040 ± 0.029 | 0.034 ± 0.038 | 0.030 ± 0.035 |
| Obese | 0.044 ± 0.046 | -0.181 ± 0.061 | -0.215 ± 0.056 |
| *p-value for trend* | 0.004 | <0.001 | <0.001 |
| Sedentary |  |  |  |
| No | -0.112 ± 0.027 | 0.183 ± 0.036 | -0.014 ± 0.033 |
| Yes | 0.034 ± 0.024 | -0.069 ± 0.031 | 0.104 ± 0.029 |
| p-value | <0.001 | <0.001 | 0.009 |

Analysis performed on 3,799 participants. 25 participants had both extreme energy intakes and lacked data for sedentary status. Results are expressed as adjusted mean ± standard error. Statistical analysis performed by analysis of variance simultaneously adjusting for all the variables listed in the table.
